# TESS 2.0—Adaptation of the German Version of the Toronto Extremity Salvage Score: Addition of an Item Regarding the Use of a Touchscreen and Keyboard in the Upper Extremity Questionnaire

**DOI:** 10.3390/jcm15020741

**Published:** 2026-01-16

**Authors:** Christoph Hofer, Leonie-Sophie Kutscha-Lissberg, Reinhard Windhager, Gerhard M. Hobusch, Carmen Trost

**Affiliations:** 1 Department of Orthopedics and Trauma Surgery, Division of Orthopedics, Medical University of Vienna, 1090 Vienna, Austria; leonie-sophie.kutscha-lissberg@meduniwien.ac.at (L.-S.K.-L.); reinhard.windhager@meduniwien.ac.at (R.W.); gerhard.hobusch@meduniwien.ac.at (G.M.H.); 2 Teaching Center, Department Assessment and Skills, Medical University of Vienna, 1090 Vienna, Austria; carmen.trost@meduniwien.ac.at

**Keywords:** Toronto Extremity Salvage Score (TESS 2.0), upper extremity function, patient-reported outcome measure (PROM), limb salvage surgery, bone and soft tissue tumors, cross-cultural adaptation, reliability and validity, German version

## Abstract

**Background/Objectives**: Limb salvage surgery is the preferred treatment for bone and soft tissue tumors. The Toronto Extremity Salvage Score (TESS) is a validated patient-reported outcome measure (PROM). However, its upper extremity section is outdated, lacking assessment of modern technology use. This study adapted TESS (creating TESS 2.0) by adding a question on touchscreen/keyboard use, based on an existing Italian version. **Methods**: Two independent translations of the new item were synthesized. Linguistic feedback was obtained from two German speakers from Germany and Switzerland. Pretesting with eight individuals refined the wording. Fourteen patients who underwent upper extremity surgery completed the TESS 2.0 twice (once in person, once at home) and the DASH questionnaire once. Reliability was assessed using Cronbach’s alpha and ICC, and validity was assessed using Spearman’s correlation between TESS 2.0 and DASH. **Results**: TESS 2.0 demonstrated excellent internal consistency (Cronbach’s alpha ≥ 0.98 at both time points). A strong, statistically significant inverse correlation existed between TESS 2.0 and DASH (r = −0.867, *p* < 0.001). Test–retest reliability was high (ICC = 0.98). **Conclusions**: German TESS 2.0 is a reliable and valid instrument for assessing upper extremity function in patients treated for bone and soft tissue tumors. Further research is needed to validate its use in postoperative follow-up and a larger, more diverse patient population.

## 1. Introduction

The state-of-the-art treatment for malignant bone and soft tissue tumors in the extremities involves a multidisciplinary approach that integrates surgery, radiation therapy, and chemotherapy, tailored to individual patient needs. This comprehensive strategy aims to achieve optimal oncologic outcomes while preserving limb function [1,2,3,4,5]. Surgical treatment is also necessary in the case of benign bone tumors with locally aggressive behavior [6]. Given this therapeutic focus on limb salvage, the accurate assessment of postoperative function and quality of life has become a central component of musculoskeletal care [7].

The Toronto Extremity Salvage Score (TESS) has been validated in multiple countries, demonstrating its reliability and applicability across diverse populations. Specifically, the TESS has been validated in Finland, Austria, and Greece, indicating its effectiveness in assessing functional outcomes for patients with soft tissue sarcomas in these regions [8,9,10,11].

A study by Janssen et al. found a discrepancy between clinician-reported and patient-reported function scores, with clinicians tending to overestimate patient function [12]. As one of the most used patient-reported outcome instruments, the TESS helps to minimize assessment bias and addresses the need for robust, patient-centered assessment in musculoskeletal oncology [13,14]. Mosor et al. further noted that especially younger patients perceive many commonly used functional assessments as outdated, with an overemphasis on basic tasks and insufficient consideration of more complex, socially relevant activities, including the use of digital technologies [15].

The Toronto Extremity Salvage Score (TESS) was developed in the 1990s, prior to the widespread adoption of touchscreen devices and keyboard-based interactions in daily activities [16]. Consequently, existing versions of the TESS may not fully capture functionally relevant limitations related to fine motor control, sustained upper extremity use, and digital interaction, thereby indicating a potential gap in content validity within the upper extremity questionnaire [16].

The TESS is particularly well-suited for the inclusion of a new item addressing touchscreen device usage in its upper extremity (UE) questionnaire, owing to its simplicity and the availability of validated translations in multiple languages. To ensure consistency and maintain the wide applicability of the TESS, we adhered to the Italian version’s approach, which explicitly incorporated this item during its validation process. This alignment supports the continued international relevance and standardized use of the TESS in diverse clinical and research contexts and strengthens the instrument’s content validity [17]. The updated version of the German TESS, referred to as German TESS 2.0, integrates contemporary considerations to better reflect modern lifestyles.

The key enhancement is the inclusion of an item addressing the ubiquitous role of touchscreen devices in daily activities, especially communication, work, and leisure. This is particularly important for individuals with upper extremity impairments whose quality of life and independence can be significantly impacted by the accessibility and usability of such technology. By incorporating this item, the German TESS 2.0 aims to provide a more comprehensive and relevant assessment of functional performance in today’s technological landscape [14].

## 2. Materials and Methods

To incorporate the new item (question 30) into the existing German version of the TESS, we conducted a psychometric single-center validation study to assess the validity, reliability, and internal consistency of this addition. The German version of the TESS has already been validated and published in March 2021. The current study aimed specifically to evaluate the inclusion of item question 30. This study received approval and underwent review by the ethics committee of the Medical University of Vienna. Prior to participation, each individual provided their informed consent by signing the designated consent form.

Between June 2022 and January 2023, patients attending the Orthopedic Tumor Outpatient Clinic in Vienna were consecutively invited to participate in this study. Eligibility criteria included the following: being 18 years or older, having been diagnosed with a malignant or benign bone or soft tissue tumor of the upper extremities, having undergone a post-surgical period of at least three months, and having no history of disease recurrence or other significant health issues. Following Rossi et al., we also included benign tumors whose surgical treatments were comparable to those of malignant tumors [17].

Patients unable to complete the questionnaire independently, such as those who did not speak German, were excluded. The German TESS 2.0 and Disabilities of the Arm, Shoulder and Hand (DASH) questionnaires were administered privately in a quiet room during the patients’ waiting time. To assess test–retest reliability, patients were instructed to complete the German TESS 2.0 a second time at home after at least one week and return the completed form to the clinic. Overall, 21 patients (Table 1) completed the initial questionnaire. Fourteen patients (67%) returned the second TESS 2.0 and were included in the test–retest analysis. To ensure full completion of the questionnaires, a systematic follow-up procedure was implemented for patients who did not return their second questionnaires within the specified timeframe. Initially, these patients were contacted by telephone, and the questionnaire was re-sent. Non-responders after two reminders were documented as not having completed the second questionnaire. The primary aim of the study was to validate and establish the reliability of the German TESS 2.0. Therefore, deviations from the evaluation time points specified on the German TESS 2.0 title page were accepted, as these were not deemed to be critical to the study’s objectives.

**Figure 1:**
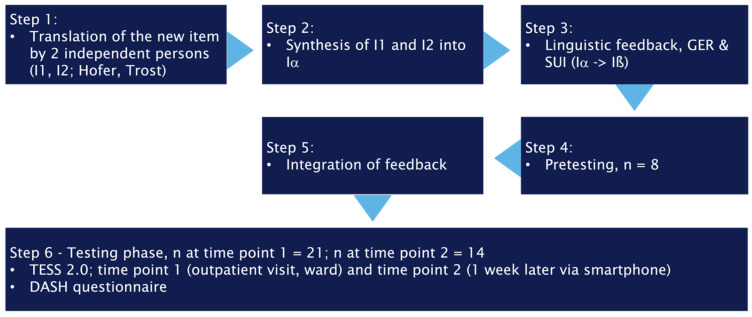
Steps in the cross-cultural adaption of question 30 on touchscreen and keyboard use in the upper extremity section.

To ensure effective cross-cultural translation and validation, adherence to five fundamental guidelines was essential [18,19]. These guidelines provide a framework for preserving the integrity and cultural relevance of the translated item. A schematic overview of these principles is shown in Figure 1.

The development of a new item on this topic followed a structured six-step approach. Initially, (1) two authors independently created a new item based on the existing Italian version of the material [17]. These independently developed items were subsequently (2) synthesized into a single cohesive version that faithfully captured the essence of the original content.

To ensure linguistic accuracy and cultural appropriateness, two German-speaking individuals, one from Germany and one from Switzerland, were consulted to provide feedback (3) on the language used in the new item. Their insights helped refine the wording to reflect regional linguistic nuances. Following the linguistic review, the new item underwent pretesting with (4) eight individuals without formal medical education. This phase was crucial for evaluating the item’s clarity and comprehensibility from a layperson’s perspective (5). Feedback interviews were conducted with these participants to gather their impressions and suggestions, which were incorporated into the final wording. After integrating (6) the feedback from the pre-testers, the German version of the questionnaire was prepared for the testing phase to evaluate its reliability, construct validity, and responsiveness.

Using this systematic approach, we aimed to develop a culturally relevant and linguistically precise item that aligns with the target audience’s needs while adhering to established cross-cultural translation guidelines.

The Toronto Extremity Salvage Score (TESS) was utilized in this study as a validated patient-reported outcome measure to assess functional status in individuals undergoing limb salvage surgery. The TESS is a patient-reported outcome measure developed to evaluate the functional status and quality of life in patients with bone and soft tissue sarcomas, particularly after limb salvage surgery. It serves as a critical tool for managing musculoskeletal oncologic conditions by assessing physical functioning and recovery. Widely used for both upper and lower extremity cases, the TESS provides valuable insights into patient recovery and satisfaction. The TESS uses a 5-point scoring scale, where a score of 100 indicates no disability and optimal functional status [9,16].

The Disabilities of the Arm, Shoulder and Hand (DASH) measurement is a widely recognized self-report tool designed to assess upper extremity function and the impact of disabilities on daily activities. Originally developed in 1996 by Hudak et al. [20], the DASH questionnaire consists of 30 items that evaluate both functional abilities and symptoms related to pain, weakness, and stiffness. Its psychometric properties have been validated across various populations and languages, demonstrating its reliability and construct validity.

The questionnaire consists of 30 items, with 23 addressing functions and seven addressing symptoms, and its domain can be analyzed using either a unidimensional or multidimensional structure, encompassing physical function, symptoms, and psychosocial aspects. The DASH utilizes a five-point scoring scale, where a score of 1 indicates no disability and optimal functional status. It consists of three parts: part one, “Functional ability”, includes 30 questions; part two, “Sport and performing arts”, and part three, “Work”, each contain four questions and are optional. For score calculation, only part one was used [21,22].

Missing data were handled differently depending on the questionnaire and according to the respective scoring rules. For the TESS, missing items were incorporated into the total score calculation as specified by the scoring algorithm. In one case, five missing responses were present and handled accordingly within the TESS computation.

For the DASH questionnaire, missing items were not incorporated into the score, as the scoring formula does not allow for this. Given the limited sample size, partially completed questionnaires were nevertheless included in the analysis. Incomplete responses occurred twice in the Garden domain and once in the Sexuality domain.

Statistical analysis was conducted to assess the validity and reliability of the German TESS 2.0, using IBM SPSS Statistics, Version 29.0 (Chicago, IL, USA) for all computations. Spearman rank correlation coefficients were calculated between the DASH part one “Functional ability” scores and the TESS 2.0 first time point scores [23,24,25].

Cronbach’s alpha assessed internal consistency [26]. In the test–retest analysis, the intraclass correlation coefficient (ICC, a two-way mixed effects model with absolute agreement, considering subjects as random effects and measurement occasions as fixed effects) was computed between baseline and repeated measures for each question’s responses and the overall German TESS 2.0 score [27]. Sample size determination followed the approach by Giraudeau and Mary for planning reproducibility studies based on the expected width of the confidence interval of the intraclass correlation coefficient. Assuming an expedited ICC of 0.89, a two-sided 95% confidence interval (α = 0.05), a desired confidence interval width of 0.23, and two repeated measurements per subject, the required sample size was 14 individuals [28]. Bland–Altman plots visually evaluated the range of discrepancies [29].

To interpret the strength of relationships, we categorized correlation coefficients and Intraclass Coefficients (ICCs) as follows: values of 0.70 or greater were considered strong, values between 0.50 and 0.70 (exclusive of 0.70) were considered moderate, and values of 0.50 or less were considered weak. This categorization aligns with established benchmarks in the literature [24,25,30]. Statistical significance was established at a *p*-value of 0.05.

## 3. Results

In a pilot project evaluating the implementation of the electronic German TESS on smartphones, younger participants reported that there was no question of difficulties in using those devices. Following consultation with the original author, Davis, we decided to align the German version with the Italian version, which includes a question about the difficulties using touchscreen devices. Consequently, we incorporated the option of using a keyboard into the wording of an additional question (number 30) in the German version:

30) Das Schreiben am Computer oder einem Touchscreen ist:

1____unmöglich;

2____extrem schwierig;

3____mäßig schwierig;

4____ein wenig schwierig;

5____überhaupt nicht schwierig.

Assessing the Psychometric Properties of the TESS 2.0

An overview of the descriptive statistics for TESS 2.0 total scores, the newly added item, and DASH is provided in the following Table 2, which presents mean, standard deviation (SD), and range (minimum–maximum). 14 patients completed both TESS 2.0 assessments and were included in the test–retest reliability analysis. DASH data were available for 20 participants, as one questionnaire (ID 21) was not returned.

Including the newly adapted item, the German TESS 2.0 upper extremity questionnaire demonstrated excellent internal consistency at both time points, with a Cronbach’s alpha of 0.98 at t1 and 0.99 at t2. This high level of internal consistency indicates that the items within the questionnaire are reliably measuring the same construct across both assessments.

**Figure 2:**
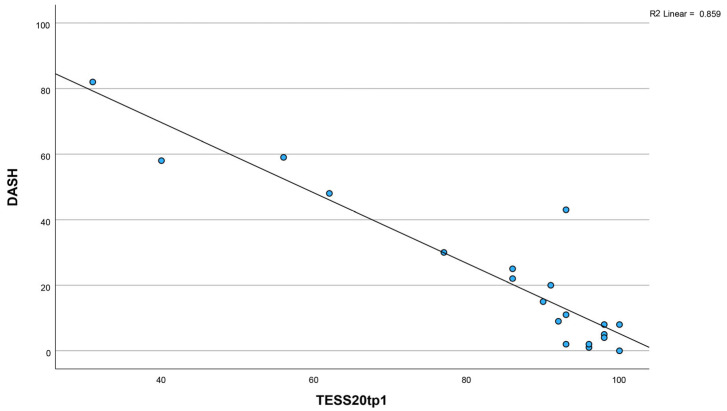
The inverse correlation refers to the mirrored scaling of both scores. German TESS 2.0: 100 = no disabilities—good function; DASH: 0 = no disabilities—good function. (Spearman’s = r = −0.867 (*p* < 0.001).

A statistically significant correlation was observed between the TESS 2.0 and the DASH, with a Spearman’s rank correlation coefficient of r = −0.867 (*p* < 0.001). The negative correlation arises because the instruments are scored inversely; higher scores reflect greater disability on one and better function on the other, as illustrated in Figure 2.

**Figure 3:**
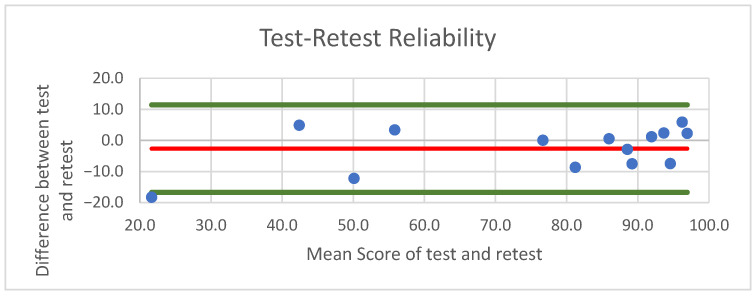
Bland–Altmann plot illustrating the agreement between TESS 2.0 scores at baseline (t1) and re-test (t2). Each dot represents one participant (n =14); the red line shows the mean difference and the green lines the 95% limits of agreement. The mean difference between measurements was −2.7 points, with a standard deviation of the differences of 7.16. The 95% limits of agreement ranged from −16.70 to 11.39, indicating high agreement and minimal systematic bias across the measurement range.

Finally, the intraclass correlation coefficient (ICC) between the baseline (t1) and re-test (t2) scores was 0.98, suggesting excellent test–retest reliability (Figure 3). This indicates that the adapted TESS 2.0 upper extremity questionnaire provides stable and consistent results over time.

## 4. Discussion

The primary aim of this study was to adapt the German Toronto Extremity Salvage Score (TESS) by integrating a novel item addressing touchscreen device use and to evaluate its psychometric properties in patients with tumors of the upper extremity. The German TESS 2.0 demonstrated excellent internal consistency (Cronbach’s alpha ≥ 0.98), high test–retest reliability (ICC = 0.98), and strong construct validity, as evidenced by a statistically significant inverse correlation with the DASH questionnaire (r = −0.867, *p* < 0.001). These findings indicate that the addition of the new item does not compromise the reliability or validity of the instrument and supports its use as a stable and robust patient-reported outcome measure.

This discussion explores the relevance and methodological rigor underpinning the implementation of the German TESS 2.0, an advancement of the German Toronto Extremity Salvage Score (TESS). This updated iteration integrates an item addressing the use of touchscreen devices within its upper extremity questionnaire, thereby reflecting the growing prominence of digital technologies in daily life.

The original TESS was designed to evaluate functional limitations in individuals with extremity tumors. The necessity for its revision stems from the rapid pace of technological development and the consequent shifts in patients’ experiences. Mosor et al. suggests that many existing questionnaires, especially those intended for younger individuals, no longer accurately reflect contemporary lifestyles [15]. Furthermore, a qualitative study by Trost et al. indicates patients’ desires for more personalized questionnaires that capture their specific needs and experiences [31]. Given the increasing prevalence of touchscreen devices, this aspect must be included in the evaluation of functional limitations.

The translation and validation of TESS across diverse languages are crucial for ensuring the comparability of the study’s findings and the instrument’s applicability in various cultural contexts. Studies by Rossi et al. and Trost et al. detail the processes of cultural adaptation and validation for the Italian and German versions of TESS, respectively [9,17]. These emphasize the importance of back-translations, cognitive debriefing, and engaging native-speaking experts. The translation of questionnaires is complicated by cultural and linguistic nuances.

The development of the German TESS 2.0 represents a significant advancement in terms of content relevance, while the present psychometric findings indicate that the integration of the new item does not adversely affect measurement reliability or validity.

The TESS was originally designed for patients with sarcomas. However, our study also included patients with benign tumors due to the comparable surgical interventions required for both. Nevertheless, a selection bias must be discussed.

Despite the small sample size due to the rarity of musculoskeletal tumors in the upper extremities, the findings should be interpreted with caution given the small sample size, although the observed psychometric properties were consistently strong. The excellent internal consistency (Cronbach’s alpha > 0.98) and test–retest reliability (ICC = 0.98) demonstrate the reliability of the German TESS 2.0. While a small sample size may limit statistical power, it is a common challenge in research on rare conditions and does not preclude meaningful interpretation of the results, but limits their generalizability. Additionally, the selected population is representative of the target group, as all patients underwent comparable surgical interventions and faced similar postoperative challenges.

The risk of inter-observer and response bias was minimized by administering the first TESS 2.0 questionnaire in person during the outpatient visit, which allowed participants to ask clarifying questions when needed, although the items were sufficiently clear that additional explanation was rarely required. For the second assessment, participants received the questionnaire to complete at home one week later, and were reminded by telephone when necessary; loss to follow-up occurred only due to nonresponse or withdrawal when the final diagnosis no longer met the inclusion criteria. The TESS 2.0 score was calculated strictly according to the original scoring instructions, as detailed in the Supplementary Materials, ensuring methodological consistency and avoiding quantitative heterogeneity in score conversion. The modest proportion of participants completed the retest (67%) may have influenced the precision of the test–retest reliability estimates, although the observed ICC remained high. Taken together, the results should be regarded as preliminary but encouraging, providing a basis for further validation in larger and more diverse cohorts.

The use of DASH as a comparison tool aligns with established methods in functional outcome assessment, but future studies could benefit from incorporating additional PROMs.

To address generalizability, further validation in larger, more diverse cohorts across different cultural and linguistic settings is recommended. Long-term studies are also suggested to assess sustained functional outcomes over time.

Implementing TESS 2.0 offers several advantages. By addressing touchscreen device usage and the patient’s viewpoint, it becomes a more relevant instrument for assessing contemporary quality of life. It also permits a more comprehensive evaluation of functional results by incorporating the social, emotional, and personal factors of recovery. The focus on patient perspectives in German TESS 2.0 fosters patient-centered care, prioritizing their needs and expectations. Finally, the information generated by the German TESS 2.0 can support clinical decision-making in treatment and rehabilitation planning.

## 5. Conclusions

In conclusion, the implementation of German TESS 2.0 is a vital step forward in improving the assessment of functional outcomes in patients undergoing limb salvage surgery for musculoskeletal tumors. The instrument’s updated design and its incorporation of patient viewpoints make German TESS 2.0 a more pertinent, comprehensive, and patient-focused tool for assessing quality of life. Including inquiries related to social support, and individual patient goals would further enhance German TESS 2.0 and make a significant contribution to enhancing care for individuals with extremity tumors.

## Figures and Tables

**Table 1:** Patient demographics. Of the twenty-one participants enrolled, four were excluded after inclusion (IDs 3, 11, 15, 16) and three did not return the T2 questionnaire despite repeated reminders.

Characteristics	Number of Patients
Overall (enrolled, completed t1 and t2)	21 (21, 14)
Age (range, mean)	25–79; 56.7
Sex (male; female)	8; 13

**Table 2:** Descriptive statistics of the instruments and the new added item.

Instrument	Mean	SD	Range (Min–Max)	N
TESS 2.0 total t1	81.7	20.5	30.8–100	21
TESS 2.0 total t2	74.7	25.6	12.5–99.2	14
New item - t1	4.5	0.8	2–5	21
New item - t2	4.3	1.1	1–5	14
DASH	23.4	23.4	0–81.7	20

## Data Availability

The data presented in this study are available on reasonable request from the corresponding author. Please contact Christoph Hofer at christoph.hofer@meduniwien.ac.at for access to the anonymized dataset.

## Supplementary Materials

The following supporting information can be downloaded at https://www.mdpi.com/article/10.3390/jcm15020741/s1. TESS 2.0_Questionnaire_GERMAN.

## Abbreviations

The following abbreviations are used in this manuscript:

DAHS	Disabilities of the Arm, Shoulder and Hand
EC	Ethics Committee
ICC	Intraclass correlation coefficient
PROMs	Patient-reported outcome measures
