# Supplementary material for: TESS 2.0—Adaptation of the German Version of the Toronto Extremity Salvage Score: Addition of an Item Regarding the Use of a Touchscreen and Keyboard in the Upper Extremity Questionnaire
Source: J Clin Med. 2026 Jan 16;15(2):741. doi: 10.3390/jcm15020741 (PMC12841948; doi:10.3390/jcm15020741)
Supplement: Supplementary file 1 [file jcm-15-00741-s001.zip › jcm-4078491-supplementary.pdf]

Toronto Extremity Salvage Score (TESS)

Erhobene Daten ☐

Instruktionen: Das ist das Deckblatt für die Einreichung des (SAQ*-F) Fragebogens. Diese Seite muss vom medizinischen Personal ausgefüllt werden (Krankenschwester, Datenmanager, Arzt, etc.) Für detaillierte Informationen siehe TESS Fragebogen.

1 ☐ Zeitpunkt (1)

1 Ausgangszustand
2 12 Monate nach Beginn der Behandlung
3 18 Monate nach Beginn der Behandlung
4 24 Monate nach Beginn der Behandlung

2 ☐ Hat der Patient den Fragebogen fertiggestellt? (2)

1 Nein (weiter bei Frage 3)
2 Ja

2A Datum wann der Patient den Fragebogen ausgefüllt hat

______-______-________(3)

3 ☐ Grund, warum der Fragebogen nicht ausgefüllt wurde (4)

0 Nicht zutreffend, Fragebogen wurde ausgefüllt
1 Patient verweigert wegen Krankheit
2 Patient verweigert aus anderem Grund, Begründung______________________
3 Patient konnte nicht kontaktiert werden
4 Institutioneller Fehler
5 Fragebogen nicht in der Landessprache des Patienten verfügbar
6 Anderer Grund, Begründung______________________
9 Unbekannt

4 ☐ Erhebungsmethode (7)

0 Nicht zutreffend (nicht ausgefüllt)
1 Bei der Visite
2 Via E-Mail
3 Telefonisch
4 Unbekannt

5 ☐ Benötigte der Patient Hilfestellung für das Ausfüllen? (8)

0 Nicht zutreffend (nicht ausgefüllt)
1 Nein
2 Ja
3 Unbekannt, wenn Hilfestellung gegeben wurde

6 ☐ Wer gab die Hilfestellung? (9)

0 Nicht zutreffend (nicht ausgefüllt, keine Assistenz)
1 Mitarbeiter
2 Familie
3 Andere,
Wer?________________(10)
9 Unbekannt

6 ☐ Ausmaß der gegebenen Hilfestellung (11)

0 Nicht zutreffend (nicht ausgefüllt, keine Assistenz)
1 Die Fragen wurden vorgelesen
2 Die Fragen wurden erklärt
3 Fragen wurden nach Antwort des Patienten angekreuzt
4 Kombination
aus:_____________(12)
5 Andere,
welche?_____________(13)
9 Unbekannt

____
Unterschrift des Ausfüllenden (14)

_____-_____-_______
Datum der Erstellung (15)

Toronto Extremity Salvage Score (TESS)
Administration des Fragebogens

Allgemeine Richtlinien

Dieser Fragebogen wurde entwickelt zur Funktionsmessung von Patienten mit physikalischen Beeinträchtigungen nach einer extremitätenerhaltenden Operation von muskuloskeletalen Tumoren. Das ist ein selbstständig auszufüllender Fragebogen.

Punktevergabe

Jede Frage dient als Maß für die Schwierigkeit zur Erfüllung der Aufgabe. Die Gesamtpunktezahl für eine Einheit bedeutet den bestmöglichen Leistungswert (d.h. 5).

Die Skala wurde dahingehend entwickelt, dass die Teilnehmer auch in einer nicht-anwendbaren Kategorie antworten können, wenn sie diese nicht in ihrer täglichen Routine ausüben. Demzufolge wird der Gesamtscore des Fragebogens standardisiert zwischen 0 – 100 berechnet:

$$\frac{\text{Summe der Gesamtpunktezahl} - \#\ \text{Items}}{\text{möglicher Punkteintervall}} \quad \text{X } 100\%$$

Summe der Itemscores = Summe der Beeinträchtigungsreaktion
Item = beantwortete Items ausgenommen der nicht beantworteten Items (NA)
möglicher Punkteintervall = (5 x #Items) – (1 x #Items)

Mail Administration

Die Fragebögen wurden per Post verschickt und obwohl keine formale Prüfung stattgefunden hat, sind die Patienten bereit und in der Lage die Fragebögen auszufüllen und die Werte liegen innerhalb der erwarteten Bereiche.

Toronto Extremity Salvage Score
(Davis, 1996)

Fragebogen für die oberen Extremitäten
Fragebogen für die unteren Extremitäten

Vom Patienten ausgefüllt zum Ausgangszeitpunkt, 12, 18 und 24 Monate nach Behandlungsbeginn.
Merke: nur der Fragebogen für die obere ODER die untere Extremität wird ausgefüllt, nicht beide.

TESS – Obere Extremität

Patienten ID#: ________________ Patienten Initials:___________________
(VN,MN,NN)

Geburtsdatum:________________ Erstellungsdatum:__________________
(Tag/Monat/Jahr) (Tag/Monat/Jahr)

Monate nach Beginn der Behandlung:
◊ Ausgangszeitpunkt
◊ 12 Monate
◊ 18 Monate
◊ 24 Monate

Seite: 1______ Weichteil
2______ Knochen

Betroffene Seite: 1_____ rechts
2_____ links

Sind Sie: 1________ Rechtshänder
2________ Linkshänder

Folgende Fragen behandeln körperliche Aktivitäten, die Sie im gewöhnlichen Alltagsleben ausführen. Neben jeder Frage findet sich eine Abstufungsskala der Schwierigkeit zur Ausführung der Aufgabe. Kreuzen Sie bitte die am ehesten zutreffende Aussage rückblickend auf den Zeitraum der **vergangenen Woche** an. Einige der körperlichen Aktivitäten werden für Sie sehr einfach sein, andere werden extrem schwierig oder sich als unmöglich erweisen.

BEISPIEL

Gemüse schälen ist:

1____unmöglich
2____extrem schwierig
3____mäßig schwierig
4____ein wenig schwierig
5____überhaupt nicht schwierig

888____Diese Fragestellung trifft auf mich nicht zu.

Sie sollten die Antwort „unmöglich" wählen, wenn die Aktivität Bestandteil Ihres üblichen Alltagslebens ist, aber nun aufgrund körperlicher Einschränkung, wie Schwäche, Steifheit und Schmerzen nicht mehr ausführbar ist. Wählen Sie die Antwort „888" aus, wenn eine der genannten Tätigkeiten in Ihrem Alltagsleben nicht vorkommt.
Kreuzen Sie bitte die am ehesten zutreffende Aussage rückblickend auf den Zeitraum der **vergangenen Woche** an.
Die folgenden Fragen behandeln etwaige Aktivitäten, welche Sie für gewöhnlich in Ihrem täglichen Leben ausüben.
Wählen Sie die Antworten, welche für Sie am besten Ihre Fähigkeit zur Ausführung der Aktivitäten beschreiben. Beziehen Sie derzeitige Einschränkungen aufgrund Ihres Armes, innerhalb der **vergangenen Woche** mit ein.

1) Eine Hose anziehen ist

1____unmöglich
2____extrem schwierig
3____mäßig schwierig
4____ein wenig schwierig
5____überhaupt nicht schwierig

888____Diese Fragestellung trifft auf mich nicht zu.

2) Schuhe zubinden ist:

1____unmöglich
2____extrem schwierig
3____mäßig schwierig
4____ein wenig schwierig
5____überhaupt nicht schwierig

888____Diese Fragestellung trifft auf mich nicht zu.

3) Socken oder Kniestrümpfe anziehen ist:

1____unmöglich
2____extrem schwierig
3____mäßig schwierig
4____ein wenig schwierig
5____überhaupt nicht schwierig

888____Diese Fragestellung trifft auf mich nicht zu.

4) Duschen ist:

1____unmöglich
2____extrem schwierig
3____mäßig schwierig
4____ein wenig schwierig
5____überhaupt nicht schwierig

888____Diese Fragestellung trifft auf mich nicht zu.

5) Meinen Oberkörper und Arme ankleiden ist:

1____unmöglich
2____extrem schwierig
3____mäßig schwierig
4____ein wenig schwierig
5____überhaupt nicht schwierig

888____Diese Fragestellung trifft auf mich nicht zu.

6) Ein Hemd / eine Bluse zuknöpfen ist:

1____unmöglich
2____extrem schwierig
3____mäßig schwierig
4____ein wenig schwierig
5____überhaupt nicht schwierig

888____Diese Fragestellung trifft auf mich nicht zu.

7) Eine Krawatte oder eine Schleife am Kragen einer Bluse / eines Hemdes zu binden ist:

1____unmöglich
2____extrem schwierig
3____mäßig schwierig
4____ein wenig schwierig
5____überhaupt nicht schwierig

888____Diese Fragestellung trifft auf mich nicht zu.

8) Make-up auflegen oder rasieren ist:

1____unmöglich
2____extrem schwierig
3____mäßig schwierig
4____ein wenig schwierig
5____überhaupt nicht schwierig

888____Diese Fragestellung trifft auf mich nicht zu.

9) Zähneputzen ist:

1____unmöglich
2____extrem schwierig
3____mäßig schwierig
4____ein wenig schwierig
5____überhaupt nicht schwierig

888____Diese Fragestellung trifft auf mich nicht zu.

10) Haare bürsten ist:

1____unmöglich
2____extrem schwierig
3____mäßig schwierig
4____ein wenig schwierig
5____überhaupt nicht schwierig

888____Diese Fragestellung trifft auf mich nicht zu.

11) Leichte Hausarbeiten wie Aufräumen und Staub wischen sind:

1____unmöglich
2____extrem schwierig
3____mäßig schwierig
4____ein wenig schwierig
5____überhaupt nicht schwierig

888____Diese Fragestellung trifft auf mich nicht zu.

12) Gartenarbeit ist:

1____unmöglich
2____extrem schwierig
3____mäßig schwierig
4____ein wenig schwierig
5____überhaupt nicht schwierig

888____Diese Fragestellung trifft auf mich nicht zu.

13) Mahlzeiten zubereiten und servieren ist:

1____unmöglich
2____extrem schwierig
3____mäßig schwierig
4____ein wenig schwierig
5____überhaupt nicht schwierig

888____Diese Fragestellung trifft auf mich nicht zu.

14) Schneiden der Mahlzeiten während dem Essen ist:

1____unmöglich
2____extrem schwierig
3____mäßig schwierig
4____ein wenig schwierig
5____überhaupt nicht schwierig

888____Diese Fragestellung trifft auf mich nicht zu.

15) Aus einem Glas trinken ist:

1____unmöglich
2____extrem schwierig
3____mäßig schwierig
4____ein wenig schwierig
5____überhaupt nicht schwierig

888____Diese Fragestellung trifft auf mich nicht zu.

16) Schwere Hausarbeiten wie Staubsaugen und das Umstellen von Möbel sind:

1____unmöglich
2____extrem schwierig
3____mäßig schwierig
4____ein wenig schwierig
5____überhaupt nicht schwierig

888____Diese Fragestellung trifft auf mich nicht zu.

17) Einkaufen gehen ist:

1____unmöglich
2____extrem schwierig
3____mäßig schwierig
4____ein wenig schwierig
5____überhaupt nicht schwierig

888____Diese Fragestellung trifft auf mich nicht zu.

18) Hantieren mit Münzen und Geldscheinen ist:

1____unmöglich
2____extrem schwierig
3____mäßig schwierig
4____ein wenig schwierig
5____überhaupt nicht schwierig

888____Diese Fragestellung trifft auf mich nicht zu.

19) Eine Einkaufstasche oder Aktenkoffer tragen ist:

1____unmöglich
2____extrem schwierig
3____mäßig schwierig
4____ein wenig schwierig
5____überhaupt nicht schwierig

888____Diese Fragestellung trifft auf mich nicht zu.

20) Eine Schachtel in ein überkopfhohes Regal stellen ist:

1____unmöglich
2____extrem schwierig
3____mäßig schwierig
4____ein wenig schwierig
5____überhaupt nicht schwierig

888____Diese Fragestellung trifft auf mich nicht zu.

21) Einen Schlüssel im Schloss umdrehen ist:

1____unmöglich
2____extrem schwierig
3____mäßig schwierig
4____ein wenig schwierig
5____überhaupt nicht schwierig

888____Diese Fragestellung trifft auf mich nicht zu.

22) Eine Tür aufdrücken oder aufziehen ist:

1____unmöglich
2____extrem schwierig
3____mäßig schwierig
4____ein wenig schwierig
5____überhaupt nicht schwierig

888____Diese Fragestellung trifft auf mich nicht zu.

23) Schreiben ist:

1____unmöglich
2____extrem schwierig
3____mäßig schwierig
4____ein wenig schwierig
5____überhaupt nicht schwierig

888____Diese Fragestellung trifft auf mich nicht zu.

24) Kleine Gegenstände aufheben ist:

1____unmöglich
2____extrem schwierig
3____mäßig schwierig
4____ein wenig schwierig
5____überhaupt nicht schwierig

888____Diese Fragestellung trifft auf mich nicht zu.

25) Meiner Arbeit nachgehen ist: (Haushalt und Beruf)

1____unmöglich
2____extrem schwierig
3____mäßig schwierig
4____ein wenig schwierig
5____überhaupt nicht schwierig

888____Diese Fragestellung trifft auf mich nicht zu.

26) Das Erfüllen meiner üblichen Arbeitsstunden ist: (Haushalt und Beruf)

1____unmöglich
2____extrem schwierig
3____mäßig schwierig
4____ein wenig schwierig
5____überhaupt nicht schwierig

888____Diese Fragestellung trifft auf mich nicht zu.

27) Meinen üblichen Freizeitaktivitäten nachzugehen ist:

1____unmöglich
2____extrem schwierig
3____mäßig schwierig
4____ein wenig schwierig
5____überhaupt nicht schwierig

888____Diese Fragestellung trifft auf mich nicht zu.

28) Sich mit Freunden und der Familie zu treffen ist:

1____unmöglich
2____extrem schwierig
3____mäßig schwierig
4____ein wenig schwierig
5____überhaupt nicht schwierig

888____Diese Fragestellung trifft auf mich nicht zu.

29) Meinen üblichen sportlichen Aktivitäten nachzugehen ist:

1____unmöglich
2____extrem schwierig
3____mäßig schwierig
4____ein wenig schwierig
5____überhaupt nicht schwierig

888____Diese Fragestellung trifft auf mich nicht zu.

30) Das Schreiben am Computer oder einem Touchscreen ist:

1____unmöglich
2____extrem schwierig
3____mäßig schwierig
4____ein wenig schwierig
5____überhaupt nicht schwierig

888____Diese Fragestellung trifft auf mich nicht zu.

1) Meine Fähigkeit alle Aktivitäten des täglichen Lebens während der letzten Woche durchzuführen, schätze ich folgendermaßen ein:

1____unmöglich
2____extrem schwierig
3____mittelmäßig schwierig
4____wenig schwierig
5____gar nicht schwierig

2) Ich würde mich selbst folgendermaßen einschätzen:

1____völlig eingeschränkt
2____stark eingeschränkt
3____mittelmäßig eingeschränkt
4____wenig eingeschränkt
5____gar nicht eingeschränkt

Bitte notieren Sie weiter unten erschwerte Aktivitäten oder etwaige andere Probleme, die Sie auf derzeitige Einschränkungen durch Ihren Arm zurückführen. Notieren Sie bitte jene, die Ihnen erwähnenswert erscheinen und in diesem Fragebogen nicht behandelt wurden.

____
____
____
____

Vergewissern Sie sich bitte, dass Sie alle Fragen beantwortet haben.

Wir möchten uns bei Ihnen für die Teilnahme sehr herzlich bedanken.

TESS – Untere Extremität

Patienten ID#: ________________ Patienten Initials:___________________
(VN,MN,NN)

Geburtsdatum:________________ Erstellungsdatum:__________________
(Tag/Monat/Jahr) (Tag/Monat/Jahr)

Monate nach Beginn der Behandlung:
◊ Ausgangszeitpunkt
◊ 12 Monate
◊ 18 Monate
◊ 24 Monate

Seite: 1______ Weichteil
2______ Knochen

Folgende Fragen behandeln körperliche Aktivitäten, die Sie im gewöhnlichen Alltagsleben ausführen. Neben jeder Frage findet sich eine Abstufungsskala der Schwierigkeit zur Ausführung der Aufgabe. Kreuzen Sie bitte die am ehesten zutreffende Aussage rückblickend auf den Zeitraum der **vergangenen Woche** an. Einige der körperlichen Aktivitäten werden für Sie sehr einfach sein, andere werden extrem schwierig oder sich als unmöglich erweisen.

BEISPIEL:
Fahrradfahren ist:

1____unmöglich
2____extrem schwierig
3____mäßig schwierig
4____ein wenig schwierig
5____überhaupt nicht schwierig

888____Diese Fragestellung trifft auf mich nicht zu.

Sie sollten die Antwort „unmöglich“ wählen, wenn die Aktivität Bestandteil Ihres üblichen Alltagslebens ist, aber nun aufgrund körperlicher Einschränkung, wie Schwäche, Steifheit und Schmerzen nicht mehr ausführbar ist. Wählen Sie die Antwort „888“ aus, wenn eine der genannten Tätigkeiten in Ihrem Alltagsleben nicht vorkommt.
Kreuzen Sie bitte die am ehesten zutreffende Aussage rückblickend auf den Zeitraum der **vergangenen Woche** an.
Die folgenden Fragen behandeln etwaige Aktivitäten, welche Sie für gewöhnlich in Ihrem täglichen Leben ausüben.

Wählen Sie die Antworten, welche für Sie am besten Ihre Fähigkeit zur Ausführung der Aktivitäten beschreiben. Beziehen Sie derzeitige Einschränkungen aufgrund Ihres Beines, innerhalb der **vergangenen Woche** mit ein.

1)Eine Hose anziehen ist:

1____unmöglich
2____extrem schwierig
3____mäßig schwierig
4____ein wenig schwierig
5____überhaupt nicht schwierig

888____Diese Fragestellung trifft auf mich nicht zu.

2)Schuhe zubinden ist:

1____unmöglich
2____extrem schwierig
3____mäßig schwierig
4____ein wenig schwierig
5____überhaupt nicht schwierig

888____Diese Fragestellung trifft auf mich nicht zu.

3)Socken oder Kniestrümpfe anziehen ist:

1____unmöglich
2____extrem schwierig
3____mäßig schwierig
4____ein wenig schwierig
5____überhaupt nicht schwierig

888____Diese Fragestellung trifft auf mich nicht zu.

4) Duschen ist:

1____unmöglich
2____extrem schwierig
3____mäßig schwierig
4____ein wenig schwierig
5____überhaupt nicht schwierig

888____Diese Fragestellung trifft auf mich nicht zu.

5)Leichte Hausarbeit, wie aufräumen oder Staub wischen ist:

1____unmöglich
2____extrem schwierig
3____mäßig schwierig
4____ein wenig schwierig
5____überhaupt nicht schwierig

888____Diese Fragestellung trifft auf mich nicht zu.

6)Gartenarbeit ist:

1____unmöglich
2____extrem schwierig
3____mäßig schwierig
4____ein wenig schwierig
5____überhaupt nicht schwierig

888____Diese Fragestellung trifft auf mich nicht zu.

7) Mahlzeiten zubereiten und servieren ist:

1____unmöglich
2____extrem schwierig
3____mäßig schwierig
4____ein wenig schwierig
5____überhaupt nicht schwierig

888____Diese Fragestellung trifft auf mich nicht zu.

8)Einkaufen gehen ist:

1____unmöglich
2____extrem schwierig
3____mäßig schwierig
4____ein wenig schwierig
5____überhaupt nicht schwierig

888____Diese Fragestellung trifft auf mich nicht zu.

9) Schwere Hausarbeit, wie staubsaugen oder Möbel verrücken, ist:

1____unmöglich
2____extrem schwierig
3____mäßig schwierig
4____ein wenig schwierig
5____überhaupt nicht schwierig

888____Diese Fragestellung trifft auf mich nicht zu.

10) In die Badewanne setzen und aus der Badewanne aufstehen ist:

1____unmöglich
2____extrem schwierig
3____mäßig schwierig
4____ein wenig schwierig
5____überhaupt nicht schwierig

888____Diese Fragestellung trifft auf mich nicht zu.

11) Ins Bett legen und aus dem Bett aufstehen ist:

1____unmöglich
2____extrem schwierig
3____mäßig schwierig
4____ein wenig schwierig
5____überhaupt nicht schwierig

888____Diese Fragestellung trifft auf mich nicht zu.

12)Von einem Stuhl aufstehen ist:

1____unmöglich
2____extrem schwierig
3____mäßig schwierig
4____ein wenig schwierig
5____überhaupt nicht schwierig

888____Diese Fragestellung trifft auf mich nicht zu.

13) Knien ist:

1____unmöglich
2____extrem schwierig
3____mäßig schwierig
4____ein wenig schwierig
5____überhaupt nicht schwierig

888____Diese Fragestellung trifft auf mich nicht zu.

14) Sich bücken, um etwas vom Boden aufzuheben ist:

1____unmöglich
2____extrem schwierig
3____mäßig schwierig
4____ein wenig schwierig
5____überhaupt nicht schwierig

888____Diese Fragestellung trifft auf mich nicht zu.

15) Treppensteigen ist:

1____unmöglich
2____extrem schwierig
3____mäßig schwierig
4____ein wenig schwierig
5____überhaupt nicht schwierig

888____Diese Fragestellung trifft auf mich nicht zu.

16) Treppen hinuntergehen ist:

1____unmöglich
2____extrem schwierig
3____mäßig schwierig
4____ein wenig schwierig
5____überhaupt nicht schwierig

888____Diese Fragestellung trifft auf mich nicht zu.

17) Autofahren ist:

1____unmöglich
2____extrem schwierig
3____mäßig schwierig
4____ein wenig schwierig
5____überhaupt nicht schwierig

888____Diese Fragestellung trifft auf mich nicht zu.

18) Im Haus umhergehen ist:

1____unmöglich
2____extrem schwierig
3____mäßig schwierig
4____ein wenig schwierig
5____überhaupt nicht schwierig

888____Diese Fragestellung trifft auf mich nicht zu.

19) Außerhalb des Hauses umhergehen ist:

1____unmöglich
2____extrem schwierig
3____mäßig schwierig
4____ein wenig schwierig
5____überhaupt nicht schwierig

888____Diese Fragestellung trifft auf mich nicht zu.

20) Sitzen ist:

1____unmöglich
2____extrem schwierig
3____mäßig schwierig
4____ein wenig schwierig
5____überhaupt nicht schwierig

888____Diese Fragestellung trifft auf mich nicht zu.

21)Eine Rampe hinauf- oder hinuntergehen ist:

1____unmöglich
2____extrem schwierig
3____mäßig schwierig
4____ein wenig schwierig
5____überhaupt nicht schwierig

888____Diese Fragestellung trifft auf mich nicht zu.

22) Aufrecht stehen ist:

1____unmöglich
2____extrem schwierig
3____mäßig schwierig
4____ein wenig schwierig
5____überhaupt nicht schwierig

888____Diese Fragestellung trifft auf mich nicht zu.

23) Vom Knien aufstehen ist:

1____unmöglich
2____extrem schwierig
3____mäßig schwierig
4____ein wenig schwierig
5____überhaupt nicht schwierig

888____Diese Fragestellung trifft auf mich nicht zu.

24) In bzw. aus einem Auto ein- oder aussteigen ist:

1____unmöglich
2____extrem schwierig
3____mäßig schwierig
4____ein wenig schwierig
5____überhaupt nicht schwierig

888____Diese Fragestellung trifft auf mich nicht zu.

25)Sex haben ist:

1____unmöglich
2____extrem schwierig
3____mäßig schwierig
4____ein wenig schwierig
5____überhaupt nicht schwierig

888____Diese Fragestellung trifft auf mich nicht zu.

26) Meiner Arbeit nachgehen ist: (Haushalt und Beruf)

1____unmöglich
2____extrem schwierig
3____mäßig schwierig
4____ein wenig schwierig
5____überhaupt nicht schwierig

888____Diese Fragestellung trifft auf mich nicht zu.

27) Das Erfüllen meiner üblichen Arbeitsstunden ist: (Haushalt und Beruf)

1____unmöglich
2____extrem schwierig
3____mäßig schwierig
4____ein wenig schwierig
5____überhaupt nicht schwierig

888____Diese Fragestellung trifft auf mich nicht zu.

28) Meinen üblichen Freizeitaktivitäten nachzugehen ist:

1____unmöglich
2____extrem schwierig
3____mäßig schwierig
4____ein wenig schwierig
5____überhaupt nicht schwierig

888____Diese Fragestellung trifft auf mich nicht zu.

29) Sich mit Freunden und der Familie zu treffen ist:

1____unmöglich
2____extrem schwierig
3____mäßig schwierig
4____ein wenig schwierig
5____überhaupt nicht schwierig

888____Diese Fragestellung trifft auf mich nicht zu.

30) Meinen üblichen sportlichen Aktivitäten nachzugehen ist:

1____unmöglich
2____extrem schwierig
3____mäßig schwierig
4____ein wenig schwierig
5____überhaupt nicht schwierig

888____Diese Fragestellung trifft auf mich nicht zu.

1) Meine Fähigkeit alle Aktivitäten des täglichen Lebens während der letzten Woche durchzuführen, schätze ich folgendermaßen ein:

1____unmöglich
2____extrem schwierig
3____mittelmäßig schwierig
4____wenig schwierig
5____gar nicht schwierig

2) Ich würde mich selbst folgendermaßen einschätzen:

1____völlig eingeschränkt
2____stark eingeschränkt
3____mittelmäßig eingeschränkt
4____wenig eingeschränkt
5____gar nicht eingeschränkt

Bitte kommentieren Sie untenstehend jegliche Aktivitäten, welche Sie schwierig fanden, oder etwaige andere Probleme die Sie mit Ihrem Bein hatten oder wichtig finden zu berichten, was nicht im Fragebogen vorkam.

____
____
____

Bitte kontrollieren Sie den Fragebogen, ob Sie auch alle Fragen beantwortet haben.

Danke, dass Sie sich Zeit genommen haben und die Fragen beantwortet haben.
